# Endoplasmic Reticulum Stress-Induced Autophagy Provides Cytoprotection from Chemical Hypoxia and Oxidant Injury and Ameliorates Renal Ischemia-Reperfusion Injury

**DOI:** 10.1371/journal.pone.0140025

**Published:** 2015-10-07

**Authors:** Bhavya B. Chandrika, Cheng Yang, Yang Ou, Xiaoke Feng, Djamali Muhoza, Alexandrea F. Holmes, Sue Theus, Sarika Deshmukh, Randy S. Haun, Gur P. Kaushal

**Affiliations:** 1 Department of Medicine, University of Arkansas for Medical Sciences, Little Rock, Arkansas, United States of America; 2 Department of Pharmaceutical Sciences, University of Arkansas for Medical Sciences, Little Rock, Arkansas, United States of America; 3 Central Arkansas Veterans Healthcare System, Little Rock, Arkansas, United States of America; 4 Department of Biochemistry and Molecular Biology, University of Arkansas for Medical Sciences, Little Rock, Arkansas, United States of America; Fondazione IRCCS Ospedale Maggiore Policlinico & Fondazione D’Amico per la Ricerca sulle Malattie Renali, ITALY

## Abstract

We examined whether endoplasmic reticulum (ER) stress-induced autophagy provides cytoprotection from renal tubular epithelial cell injury due to oxidants and chemical hypoxia *in vitro*, as well as from ischemia-reperfusion (IR) injury *in vivo*. We demonstrate that the ER stress inducer tunicamycin triggers an unfolded protein response, upregulates ER chaperone Grp78, and activates the autophagy pathway in renal tubular epithelial cells in culture. Inhibition of ER stress-induced autophagy accelerated caspase–3 activation and cell death suggesting a pro-survival role of ER stress-induced autophagy. Compared to wild-type cells, autophagy-deficient MEFs subjected to ER stress had enhanced caspase–3 activation and cell death, a finding that further supports the cytoprotective role of ER stress-induced autophagy. Induction of autophagy by ER stress markedly afforded cytoprotection from oxidants H_2_O_2_ and tert-Butyl hydroperoxide and from chemical hypoxia induced by antimycin A. In contrast, inhibition of ER stress-induced autophagy or autophagy-deficient cells markedly enhanced cell death in response to oxidant injury and chemical hypoxia. In mouse kidney, similarly to renal epithelial cells in culture, tunicamycin triggered ER stress, markedly upregulated Grp78, and activated autophagy without impairing the autophagic flux. In addition, ER stress-induced autophagy markedly ameliorated renal IR injury as evident from significant improvement in renal function and histology. Inhibition of autophagy by chloroquine markedly increased renal IR injury. These studies highlight beneficial impact of ER stress-induced autophagy in renal ischemia-reperfusion injury both *in vitro* and *in vivo*.

## Introduction

Autophagy is a dynamic process of degradation of long-lived proteins, cellular macromolecules, and intracellular organelles by the lysosomes [1,2]. This process involves formation of a double-membrane structure known as an autophagosome, which sequesters and delivers the cytoplasmic cargo to the lysosome for degradation; subsequently, the degraded products are recycled to synthesize new proteins and organelles. The molecular machinery that drives the formation of autophagosomes is composed of evolutionarily conserved Atg (**A**u**T**opha**G**y related) proteins originally identified in yeast [3,4]. Under normal physiological conditions, minimal basal autophagy occurs to maintain cellular homeostasis. Under pathological conditions autophagy is induced in response to stress conditions including cell starvation, hypoxia, and oxidant injury [3,5]. In stressed cells elevated autophagy degrades damaged material to produce metabolic substrates and bioenergetic needs of the cell. Thus, under stress conditions, autophagy induction is generally considered to play an adaptive role that promotes cell survival [5,6]. However, under some circumstances excessive and impaired autophagy can result in cell death [5,7,8,9].

In the endoplasmic reticulum (ER) lumen, newly synthesized proteins fold prior to their exit from the ER toward the various compartments of the cells and extracellular space. In response to adverse conditions in the ER, proteins are incorrectly folded and premature misfolded proteins are unable to exit from the ER lumen and accumulate in the ER resulting in ER stress [10,11]. Cells respond to ER stress by activating a highly conserved unfolded protein response (UPR) to facilitate repair toward re-establishing normal ER function [12]. The UPR involves activation of the ER transmembrane proteins inositol requiring–1α (IRE1), PKR-like ER kinase (PERK), and activating transcription factor–6 (ATF6) [11,12], which can trigger both pro-survival and pro-death pathways.

ER stress is known to induce autophagy in mammalian cells [13,14] including renal epithelial cells [15,16]. In renal proximal tubular epithelial cells, ER stress inducers tunicamycin or brefeldin A significantly increased LC3-II suggesting induction of autophagy [15,16]. However, it is not known whether ER stress-induced autophagy provides pro-survival or pro-death role in renal cells. Previous studies have reported that prior ER stress-induced UPR is capable of conferring cytoprotection from renal injury in *in vitro* and *in vivo* models of acute kidney injury (AKI) [17,18]. Heterozygous mutant-Grp78 mice developed tubulo-interstitial lesions, caspase–12 activation, and tubular apoptosis with aging [19]. Tunicamycin-induced ER stress increased the level of Grp78 and afforded protection against renal ischemia-reperfusion (IR) injury [20]. In a related study, the pharmacological compound Bix that activates UPR also attenuated renal IR injury [21]. *In vitro* studies have also demonstrated that cultured renal tubular epithelial cells (RTECs) pretreated with the ER stress inducer tunicamycin were significantly resistant to oxidative stress [22,23]. Another study demonstrated that ER stress preconditioning protected renal cells from cytotoxicity of clinically relevant nephrotoxins [24]. In an ATP-depletion model in cultured RTECs, prior activation of ER stress significantly reduced cellular injury due to antimycin A-induced chemical hypoxia-mediated ATP depletion [25,26]. However, the mechanism underlying the cytoprotection by ER stress or the role of ER stress-induced autophagy in cytoprotection from renal ischemia-reperfusion (IR) is not known. In view of the above findings, we tested whether activation of ER stress-induced autophagy is able to confer protection against subsequent oxidant and chemical hypoxia-induced cell death *in vitro* and from renal IR injury *in vivo*. For *in vitro* studies, cytoprotection by ER stress-induced autophagy against oxidants H_2_O_2_ and tert-Butyl hydroperoxide (TBHP), and ATP depletion induced by antimycin A was tested because these events are known to be associated with the pathophysiology of IR-induced AKI.

## Materials and Methods

### Cell culture and reagents

LLC-PK1 cells (porcine kidney proximal tubule epithelial cells) purchased from the ATCC were grown in M199 medium (Gibco) supplemented with 5% (v/v) heat-inactivated fetal bovine serum, 100 U/mL penicillin and 100 μg/mL streptomycin (all from Invitrogen, Carlsbad, CA) at 37°C in a humidified atmosphere containing 5% CO_2_. ATG5 (-/-) and wild-type MEFs were obtained from the RIKEN BioResource Center (Ibaraki, Japan) and maintained in 10% Dulbecco’s Modified-Eagle Medium (DMEM). Tunicamycin, thapsigargin, 3-methyladenine (3-MA), wortmannin, chloroquine, H_2_0_2_, and TBHP were obtained from Sigma. The caspase 3/7 substrate Asp-Glu-Val-Asp-aminomethyl coumarin (DEVD-AMC) was purchased from Peptide International. ER stress signaling sampler kit (Cat# 9956), mTOR signaling sampler kit (Cat# 9862S), and antibodies to cleaved caspase–3 (Cat # 9661), Atg5 (Cat # 2630), Atg12 (Cat # 4180), and LC3 (Cat # 3868) were purchased from Cell Signaling Technology (Danvers, MA). Antibodies to beclin–1 (Cat # 612112) and p62 (Cat # 610832) were from BD-Bioscience (San Diego, CA) and antibodies to β-actin (Cat # sc1616-R) were from Santa Cruz Biotechnology (Santa Cruz, CA).

### Animals, renal IR, and administration of the drugs

Animal studies were performed in strict accordance with the recommendation in the Guide for the Care and Use of Laboratory Animals of the Institute of National Health. The protocol for these studies was approved by the Animal Care and Use Committee (ACUC) of the Central Arkansas Veterans Healthcare System (PHS Assurance Number: A3509-01, protocol approval number: ACUC 3-10-3), and also by the CAVHS Safety and Research and Development Committee of the Central Arkansas Veterans Healthcare System. Ten-week-old C57BL/6 male mice were purchased from Jackson Labs. The renal ischemia-reperfusion model was developed essentially as described previously [27]. Kidneys of anaesthetized animals were exposed under sterile conditions through a midline abdominal incision. After the kidneys were decapsulated, the renal hilum was clamped for 45 min on both sides with a vascular clamp to induce ischemia. Ischemia of the kidneys was confirmed by visualization of color change of the kidney parenchyma. The kidneys were then internalized with the clamps in place. The abdomen was covered with gauze moistened in phosphate-buffered saline (PBS), and the mice were maintained at 37°C using a warming pad. Following 45 min of ischemia, the clamps were released and kidneys were again returned to their usual locations. The surgical incision was closed using a 4–0 suture. Sham-operated animals that served as control animals were subjected to the same surgical procedure except the renal pedicles were not clamped. During the course of surgery, volume depletion was prevented by administration of ~1 ml of saline into the peritoneal cavity. Postoperatively, the animals were maintained in a veterinary medical recovery unit warmed to 34°C. Oxygen was available if needed followed by recovery in the intensive care unit. After recovery from surgery, the mice were returned to their cages and allowed free access to food and water. Tunicamycin was dissolved in 70% saline + 30% DMSO and was administered intraperitoneally 2 days prior to ischemia at a dose of 1 mg/kg b.w. The control mice were administered with the corresponding vehicle in the same manner. Chloroquine was dissolved in water and administered intraperitoneally one hour before the surgery at a dose of 50 mg/kg b.w. Kidneys were harvested one day after surgery for histology and immunohistochemistry. Blood was collected for blood urea nitrogen (BUN) and serum creatinine level determinations. BUN and creatinine were determined using a diagnostic kit from International Bio-Analytical Industries Inc. (Boca Raton, FL, USA).

### Histology

Kidney tissue was fixed in phosphate-buffered 4% formalin (pH 7.4) for 24 h and then embedded in paraffin. Sections were cut into 5 μm-thick slices and used for staining with hematoxylin and eosin (H&E) and Periodic acid-Schiff (PAS).

### Immunofluorescence detection of LC-3-II-labeled autophagosomes in kidney sections

Kidney tissue was fixed in phosphate-buffered 4% formalin and paraffin embedded. Deparaffinized 5 μm sections were incubated overnight with a polyclonal rabbit anti-LC3 antibody (Novus Biologicals, Cat # NB-100-2220) followed with anti-rabbit Alexafluor-488-labeled secondary antibody (Molecular Probes) and pictures recorded using an Olympus BX51 fluorescence microscope at 40x magnification.

### GFP-LC3 transfection and imaging for scoring autophagy induction

LLC-PK1 cells were seeded on glass chamber wells and grown to 70% confluence. Cells were transfected using a GFP-LC3 Baculovirus vector system (Invitrogen) for 16 hours as per the manufacturer’s protocol. The transfected cells were treated with either 1 μg/ml tunicamycin or 100 nM thapsigargin for various time points and then the cells were fixed and permeabilized with acetone-methanol (1:1, v:v) for 5 minutes. Images of GFP-LC3 fluorescence were captured at 40 x magnifications using an Olympus BX51 fluorescence microscope equipped with a CCD camera (U-TV0.63XC). The filter configuration used for EGFP includes excitation filter of 470/30 and emission filter of 520/40 and the images were analyzed with DP manager software.

### Measurement of apoptosis by FACS

Cells were seeded on 6-well plates and after subsequent treatment the cells were harvested by trypsinization and washed with PBS followed by fixing and permeabilization with 70% ethanol for 30 minutes at 4°C. After washing the cells with ice-cold PBS, the cells were pelleted by centrifugation and incubated with 50 μg of RNase A in 250 μl of PBS for 1h at 37°C. The cells were stained with 10 μg of propidium iodide for 5 minutes in the dark and the DNA content of single cells was analyzed by FACS.

### Western blot analysis

Following various treatments, cells were harvested by scraping and lysates were prepared as described previously [28]. Protein estimation was done by Bradford assay and 100 μg of protein samples were resolved on 12 or 15% SDS-polyacrylamide gels using a Bio-Rad electrophoresis unit. Proteins were electrophoretically transferred to PVDF membranes (Bio-Rad) for 2 h at 100 V and the membranes were blocked with 5% blocking buffer for an hour. Membranes were incubated overnight at 4°C with specific primary antibodies, as noted, washed thoroughly, then incubated with corresponding secondary antibodies conjugated with HRP. The blots were then developed with the ECL reagent (Fisher Scientific) as previously described. Protein levels were quantified by densitometry using Quantity One software (Bio-Rad).

### Caspase–3/7 activity assay

Spectrofluorometric assays for caspase–3/7 were carried out as previously described [28]. In brief, cells were seeded in 60-mm dishes and various treatments were performed as indicated when the cells were 70% confluent. Cells were harvested by scraping, collected by centrifugation, and pellets washed in cold PBS and lysed with lysis buffer (Cell Signaling Technology) at 4°C. Cell lysates (25 ug total protein) were used to determine caspase–3/7 activity using 10 μM amino-4-methylcoumarin (AMC)-tagged DEVD (DEVD-AMC) substrate as described previously [28]. Spectrofluorometric readings were obtained with a Perkin Elmer plate reader using an excitation wavelength of 380 nm and an emission wavelength of 460 nm.

### LDH release assay

Quantitative determinations of cell cytotoxicity induced by oxidative stress inducers H_2_0_2_ and TBHP were assessed using an LDH release assay (Cytotoxicity Detection Kit^PLUS^, Roche Applied Science, Indianapolis, IN) according to the manufacturer’s instructions. For various treatments, cells were seeded in 96-well plates at a seeding density of 10,000 cells per well. After 24 hours, cells were given the indicated treatments in phenol red-free complete medium. Assay reagents were added at the end of each time points and incubated for 30 min before the reaction was terminated with the supplied stop reagent. The colorimetric readings were taken at 590 nm in a spectrometer.

### DAPI staining

Cells were grown to 80% confluence on glass coverslips in six-well plates and treated with the ER stress inducer tunicamycin (1μg/ml) for 24h. Following the treatment, cells were washed in PBS and fixed in 2% paraformaldehyde in PBS for 15 min. DAPI (1.5 μg/ml) staining for 5 min was used to detect fragmented and condensed nuclei as well as cell shrinkage, as morphological feature of apoptosis. Images were captured using an Olympus microscope equipped with a digital camera. Percent apoptosis for each treatment was quantified from 4 independent experiments.

### RNA interference for beclin–1 and Atg5

LLC-PK1 cells were seeded in a six-well plate with 5% FBS in M199 medium without antibiotics. Beclin siRNA (sc–29798) and Atg5siRNA (sc–41446) were obtained from Santa Cruz Biotechnology. Cells at 70% confluence were untreated (control) or transfected with the corresponding siRNA pools using Lipofectamine 2000 (Invitrogen) according to the manufacturer’s instructions. Twenty-four hours after transfection, cells were treated as indicated and a portion of the samples were collected to analyze gene silencing by Western blot analysis.

### Statistical analyses

Results are reported as mean ± SEM. Comparison between values were assessed for significance by using one-way analysis of variance (ANOVA) with Bonferroni’s post hoc test for multiple comparisons (GraphPad Prism, version 5.04). A level of *P* < 0.05 was accepted as statistically significant.

## Results

### Induction of ER stress induces UPR and autophagy in renal tubular epithelial cells

**Fig 1:**
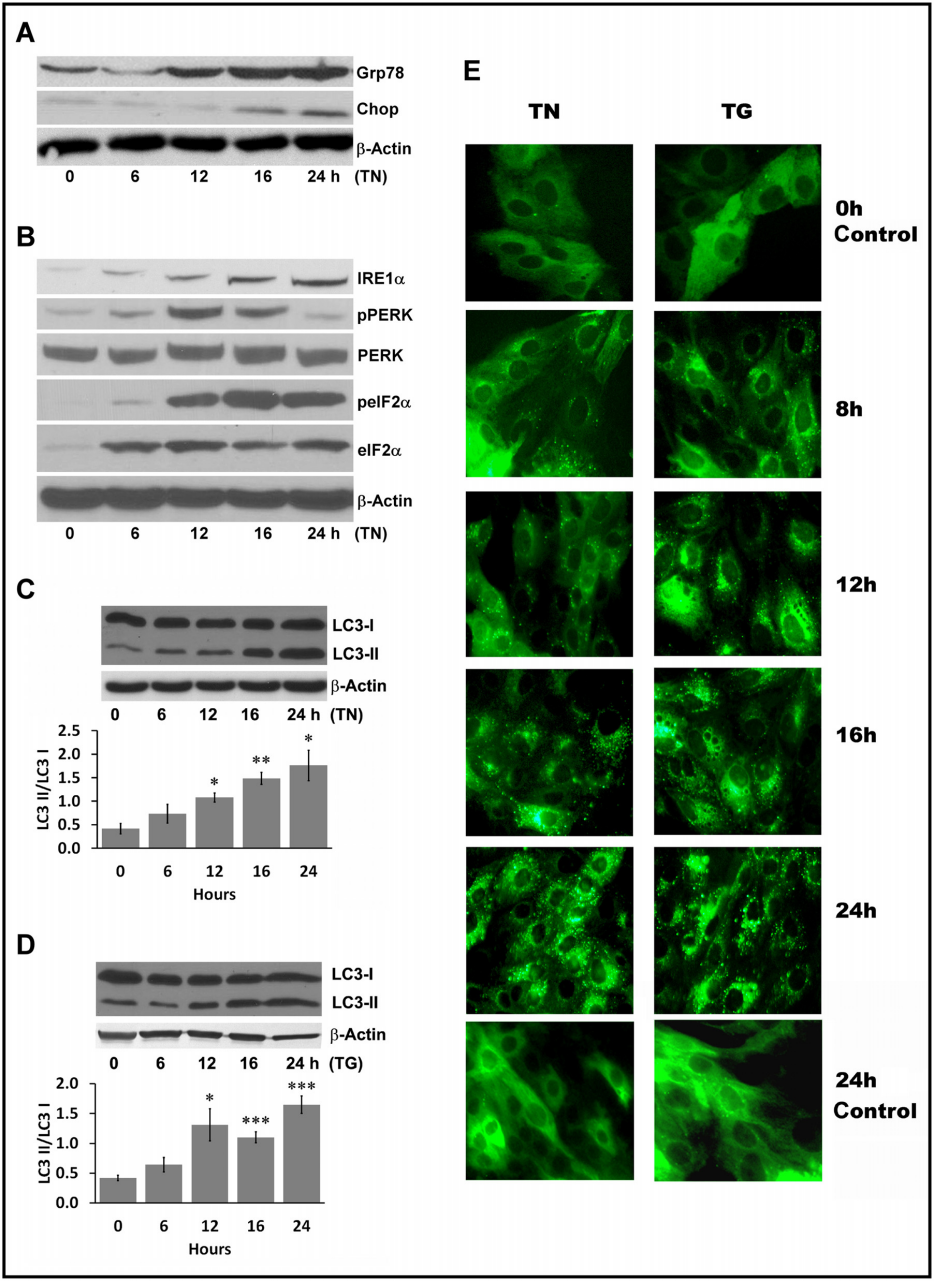
Induction of ER stress, UPR response, and autophagy by the pharmacological ER stress inducers tunicamycin and thapsigargin. **A.** LLC-PK1 cells were treated with 1 μg/ml tunicamycin (TN) for varying lengths of time as indicated. Cell lysates (100 μg protein per lane) were analyzed by western blot with antibodies specific to Grp78 and Chop to monitor the induction of the ER stress response. β-Actin served as a loading control. **B.** LLC-PK1 cells were treated with 1 μg/ml tunicamycin (TN) for varying lengths of time as indicated and cell lysates were immunoblotted for IRE1, pPERK, PERK, peIF2α, and eIF2α. β-Actin served as a loading control. **C and D.** LLC-PK1 cells were treated with (C) 1μg/ml tunicamycin (TN) or (D) 100 nM thapsigargin (TG) for varying times as indicated and autophagy induction was analyzed by monitoring LC3-I to LC3-II conversion by western blot. β-Actin served as a loading control. Bar graphs depict ratio of LC3 II/LC3 I quantified by densitometry. *P <0.05 compared with control untreated cells (0 h). **P <0.01 compared with control untreated cells (0 h). ***P <0.001 compared with control untreated cells (0 h). **E.** LLC-PK1 cells were grown on coverslips and transient transfection was carried out with a Baculovirus GFP-LC3 vector for 16 h. Treatments with 1 μg/ml tunicamycin (TN) or 100 nM thapsigargin (TG) were performed for different time points as indicated. Cells were fixed after each time points and images were captured using an Olympus fluorescent microscope. Representative images reveal increased punctate formation over treatment time.

Treatment of LLC-PK1 cells with tunicamycin, an inducer of ER stress that inhibits N-linked protein glycosylation, elicited the unfolded protein response (UPR) as revealed by increased expression of the ER stress protein Grp78 in a time-dependent manner (Fig 1A). Increased expression of Chop was observed but it was far less than Grp78. The induction of UPR was also confirmed by UPR-mediated alterations of the ER transmembrane proteins including increased expression of IRE1 and phosphorylation of PERK and IF2α (Fig 1B). Concomitantly with tunicamycin-induced ER stress proteins, conversion of LC3-I to its lipidated form, LC3-II, was significantly increased (Fig 1C). Another ER stress inducer, thapsigargin, that inhibits ER Ca^2+^ ATPase, also triggered LC3-II formation in a similar manner (Fig 1D). The conversion of LC3-I to LC3-II in response to ER stress was further examined by transfection of LLC-PK1 cells with GFP-LC3. In transfected cells, the punctate dots of GFP-LC3-II, characteristic of autophagy, was markedly increased in response to tunicamycin and thapsigargin (Fig 1E). In contrast, control transfected cells without exposure to ER stress treatment showed a diffuse distribution of green fluorescence (Fig 1E). Taken together, these studies supported the notion that autophagy is activated in response to ER stress in renal proximal tubular cells.

### Inhibition of ER stress-induced autophagy accelerates caspase activation and apoptotic cell death

**Fig 2:**
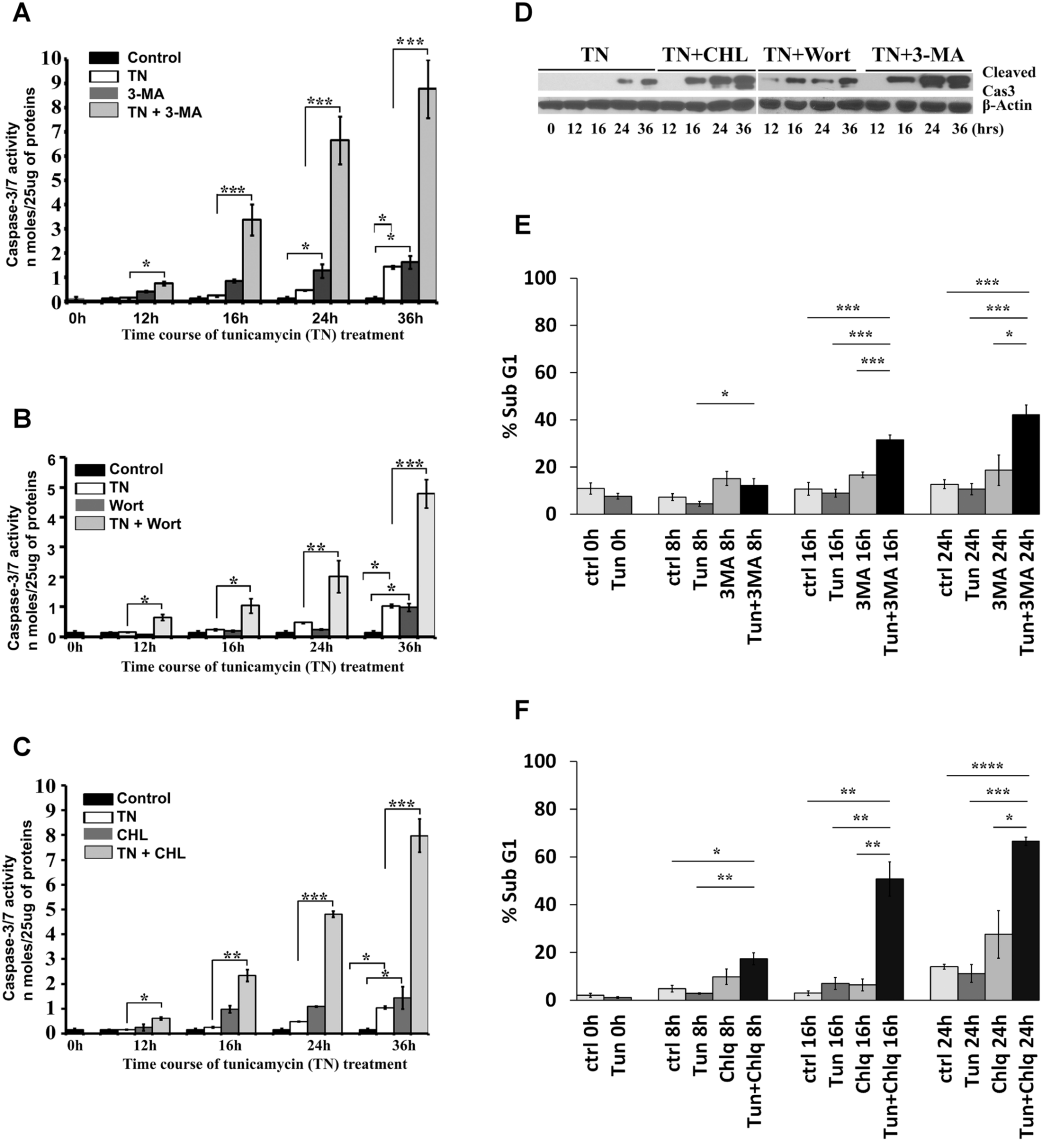
Inhibition of ER stress-induced autophagy by pharmacological inhibitors triggered caspase–3/7 activation and apoptotic cell death in LLC-PK1 cells. **A, B, and C.** LLC-PK1 cells were treated with the ER stress inducer tunicamycin (TN) for 12 h before treatment with the pharmacological autophagy inhibitors 10 mM 3-MA (**A**), 200 nM wortmannin (Wort) **(B),** or 50 μg/ml chloroquine (CHL) (**C**) for varying lengths of time as specified. Cell lysates (25 μg protein) were used for spectrofluorimetric caspase–3/7 activity assays. Control is untreated cells. Results are presented as mean±SEM of three independent experiments. *P< 0.05 compared with control or tunicamycin-treated cells. **P <0.01 compared with tunicamycin-treated cells. ***P <0.001 compared with tunicamycin-treated cells. **D.** LLC-PK1 cells were treated with the ER stress inducer tunicamycin (TN) without and with the indicated autophagy inhibitors. Cell lysates (50 μg protein) were processed for western blots to detect active caspase–3 using a cleaved caspase-3-specific antibody. β-actin served as a loading control. **E and F.** LLC-PK1 cells were treated with the ER stress inducer tunicamycin (1 μg/ml) for 12 h before treatment with 10 mM 3-MA (**E**) or 50 μg/ml chloroquine (**F**) for varying lengths of time as specified. The percentage of apoptotic cells under these treatments were determined by FACS analysis using the propidium iodide staining method. Results are represented as mean±SEM of three independent experiments. *P< 0.05 compared with control or tunicamycin-treated cells. **P <0.01 compared with tunicamycin-treated cells. ***P <0.001 compared with tunicamycin-treated cells.

The inhibitors of class-III phosphoinositide (PI) 3-kinase, 3-MA or wortmannin, block the formation of autophagy [29,30]. Also the lysosomotropic agent chloroquine that inhibits lysosomal function [31,32] inhibits autophagy by impairing autophagic flux. Thus we examined the effect of the autophagy inhibitors 3-MA, wortmannin, and chloroquine in ER stress-induced caspase activation and cell death. Inhibition of autophagy by 3-MA (Fig 2A), wortmannin (Fig 2B), and chloroquine (Fig 2C) promoted ER stress-induced caspase–3/7 activity (cleavage of DEVD-AMC) in a time-dependent (0–36 h) manner in LLC-PK1 cells. The autophagy inhibitors alone affected caspase–3/7 activation but this effect was far less than when used with tunicamycin (Fig 2A, 2B and 2C). Since the activation of caspase–3 and -7 was determined together in this assay (they utilize the same DEVD-AMC substrate), we used an antibody specific to cleaved caspase–3 that identified only caspase–3 activation but not caspase–7 activation by western blot. The proteolytic processing of procaspase–3 resulted in the formation of a 17-kDa subunit of active caspase–3 that was detected as early as 12 hours following treatment of the cells with autophagy inhibitors in the presence of tunicamycin (Fig 2D). These data suggest that inhibition of the autophagy pathway results not only in earlier activation of executioner caspase–3 but also accelerates this activation at later periods in ER stress-induced injury to RTECs. The activation of caspase–3/7 by autophagy inhibition was also reflected by increased apoptosis. Inhibition of autophagy by 3-MA (Fig 2E) and chloroquine (Fig 2F) also increased ER stress-induced apoptotic cell death. Representative flow cytometry data for each treatment and time point are shown in S1 Fig. Under similar conditions, we also examined whether inhibition of ER stress-induced autophagy results in necrotic cell death. Inhibition of tunicamycin-induced autophagy with 3-MA or wortmannin did not result in significant LDH release (data not shown).

**Fig 3:**
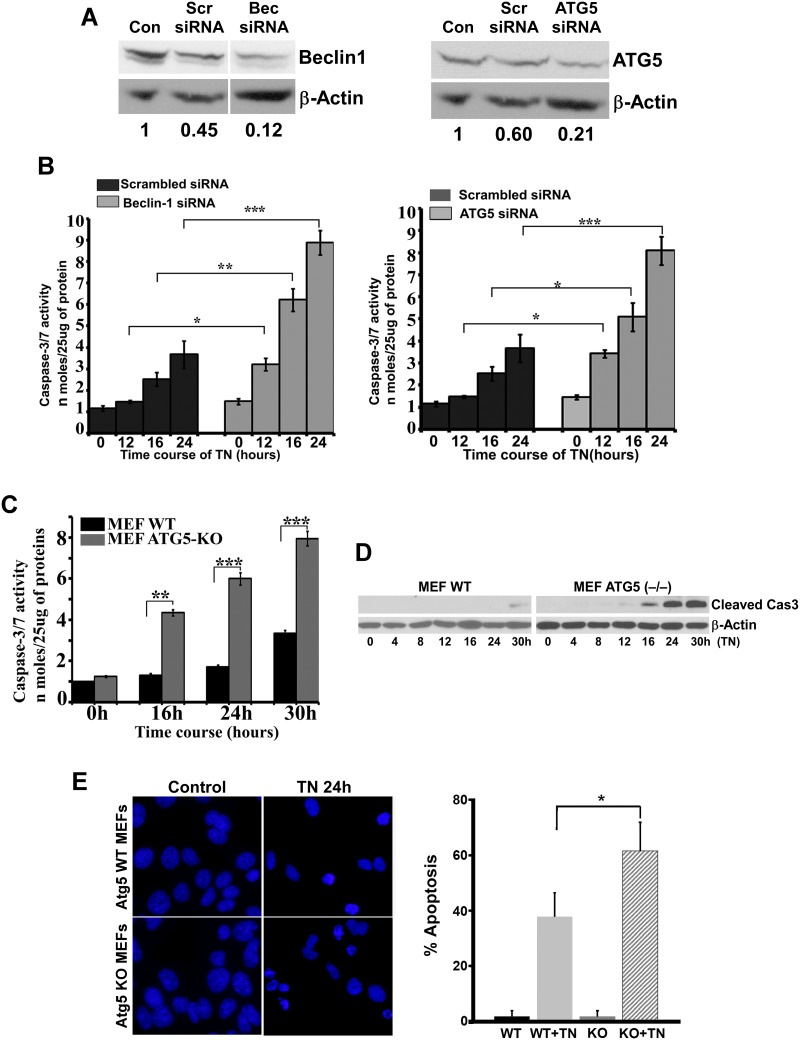
Silencing of Atg5 and beclin–1 by siRNA in renal cells and Atg5-KO MEFs triggered caspase activation upon tunicamycin treatment. **A.** LLC-PK1 cells were untreated (Con) or transfected with *Atg5* or *beclin–1* siRNAs or scrambled siRNA for 24 h as described in Materials and Method section and the cell lysates were immunoblotted for Atg5 and beclin–1. Protein levels were quantified by densitometry. siRNA-mediated silencing was evaluated as the ratio of beclin or Atg5 to β-Actin relative to untreated (Con) cells set to 1. The relative ratio for each treatment is indicated below each lane. **B**. LLC-PK1 cells silenced with Atg5 or beclin–1 siRNAs were treated with 1μg/ml tunicamycin for 12h, 16h, and 24h and caspase–3/7 activity assays were performed using a fluorogenic substrate. Results represented as mean±SEM of three independent experiments. *P < 0.05, **P<0.01, and ***P <0.001 shown are compared with scrambled siRNA-treated cells. **C.** Wild-type and Atg5-KO MEFs were treated with the ER stress inducer tunicamycin (1 μg/ml) for 16h, 24h, and 30h. Cell lysates (25 μg protein) were taken for spectrofluorimetric caspase–3/7 activity assays. Control is vehicle-treated cells. Results are presented as mean±SEM of three independent experiments. *P < 0.05, **P<0.01, and ***P <0.001 shown are compared with WT tunicamycin-treated MEFs. **D.** Wild-type and Atg5-KO MEFs were treated with the ER stress inducer tunicamycin (1μg/ml) for 16h, 24h, and 30h. Cell lysates (50 μg protein) were used for western blot using a specific antibody to active caspase–3. β-actin was used as a loading control. The results shown are representative of three independent experiments. **E.** Wild-type and Atg5-KO MEFs were treated with the ER stress inducer tunicamycin (1μg/ml) for 24h. Fragmented and condensed nuclei as well as cell shrinkage, as morphological feature of apoptosis, were measured using DAPI staining in four independent experiments. Quantification of apoptosis is shown (right). *P < 0.05 shown is compared with WT tunicamycin-treated MEFs.

We next used genetic approaches to inhibit autophagy and examined whether inhibition of autophagy afforded cell survival or cell death. We first used siRNAs targeting *beclin–1* and *atg5* for specific inhibition of autophagy. Transfection with siRNAs specific for *beclin–1* and *Atg5* markedly reduced beclin–1 and Atg5 expression in LLC-PK1 cells (Fig 3A). Down-regulation of beclin–1 and Atg5 using respective siRNA (Fig 3B) resulted in enhanced tunicamycin-induced activation of caspase–3/7 (Fig 3B). Beclin–1 and Atg5 inhibition also accelerated tunicamycin-induced cell death as revealed by an MTT assay (data not shown). We also used Atg5 (-/-) MEFs to further confirm the effect of ER stress on caspase–3 activation and cell death in autophagy-deficient cells. Upon treatment with the ER stress inducer tunicamycin, Atg5 (-/-) MEFs exhibited enhanced activation of caspase–3 (Fig 3C and 3D) and increased apoptosis (Fig 3E) compared to the wild-type MEFs, suggesting that autophagy provided a survival role against ER stress.

### ER stress-induced autophagy provides protection from oxidant injury and ATP depletion in LLC-PK1 cells

**Fig 4:**
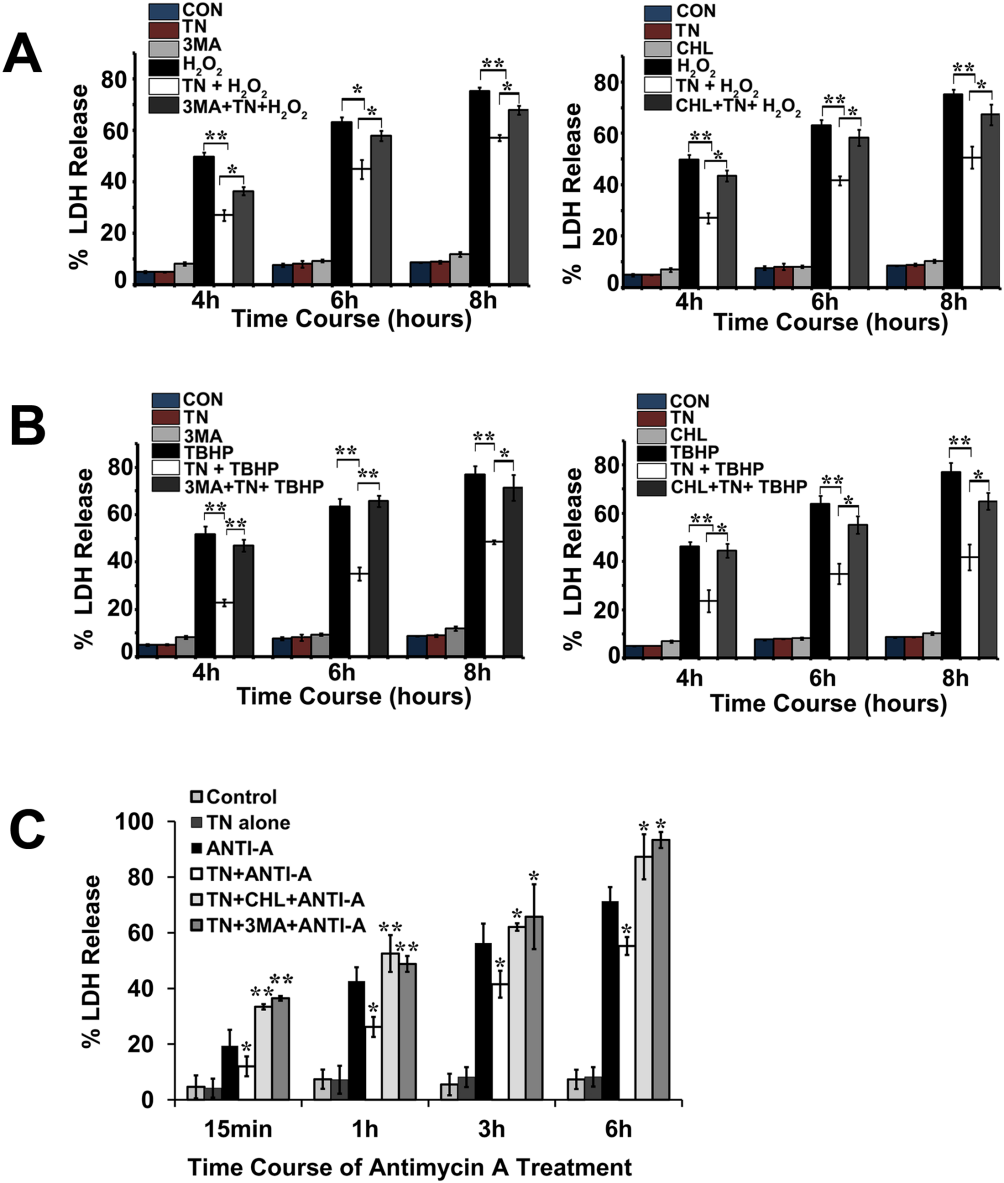
Tunicamycin pretreatment protects renal epithelial cells from oxidant injury and chemical hypoxia and protection is reversed when treated with autophagy inhibitors. **A.** LLC-PK1 cells were pretreated with 1 μg/ml tunicamycin (TN) for 12 h before a one hour treatment with 10 mM 3-MA (left) or 50 μg/ml chloroquine (CHL) (right) followed by 200 μM H_2_0_2_ for different time durations as indicated. LDH release assays were carried out as an indicator of cytotoxicity. Results represent mean±SEM of four independent experiments. *P < 0.05 compared with tunicamycin +H_2_0_2_-treated cells. **P < 0.01 compared with H_2_0_2_-treated cells. **B.** LLC-PK1 cells were pretreated with 1 μg/ml tunicamycin (TN) for 12 h before a one hour treatment with 10 mM 3-MA (left) or 50 μg/ml chloroquine (CHL) (right) followed by 50 μM TBHP for different time durations as indicated. LDH release assay was carried out as an indicator of cytotoxicity. Results represent mean±SEM of four independent experiments. *P <0.05 compared with TBHP+tunicamycin-treated cells. **P < 0.01 compared with TBHP or tunicamycin+TBHP-treated cells. **C.** LLC-PK1 cells were pretreated with 1 μg/ml tunicamycin (TN) for 12 h before a one hour treatment with 10 mM 3-MA or 50 μg/ml chloroquine followed by 10 μM antimycin A (ANTI-A)for time durations indicated. LDH release assay was carried out as an indicator of cytotoxicity. Results are expressed as mean±SEM of four different experiments. *P<0.05 compared with antimycin A or antimycin + TN-treated cells. **P<0.01 compared with TN+antimycin A-treated cells.

Previous studies have shown that prior induction of ER stress rendered RTECs resistant to cell death induced by oxidant injury [22,23] and ATP depletion [25]. We first examined the mode of cell death in LLC-PK1 cells exposed to H_2_O_2_, TBHP and antimycin A. We found that in response to oxidants and ATP depletion, LLC-PK1 cells died predominantly by necrotic cell death (cell viability detected by LDH release) and apoptosis in these cells was minimal as detected by DAPI staining (data not shown). Previous studies have also shown that LLC-PK1 cells in response to H2O2 [33,34] and ATP depletion [25] primarily undergo necrotic cell death. We have tested whether ER stress-induced autophagy provide cytoprotection of RTECs from oxidant injury and ATP depletion. As shown in Fig 4, prior ER stress induced by tunicamycin provided significant protection in cells from oxidant-induced cytotoxicity caused by H_2_O_2_ (Fig 4A) and TBHP (Fig 4B), as well as chemical hypoxia (ATP depletion)-mediated cytotoxicity caused by antimycin A. (Fig 4C). Inhibition of autophagy by 3-MA (Fig 4A, left) or chloroquine (Fig 4A, right) disrupted the ability of prior ER stress to confer protection from H_2_O_2_. Similarly, ER stress was unable to protect cells from TBHP following inhibition of autophagy by 3-MA (Fig 4B, left) or chloroquine (Fig 4B, right). Treatments with H_2_O_2_ and TBHP were done up to 8h and beyond this time majority of the cells did not survive. Also, upon autophagy inhibition, ER stress did not confer protection from ATP depletion resulting from antimycin A treatment (Fig 4C). These studies support the notion that prior autophagy activation played an important role in cytoprotection of renal tubular epithelial cells against oxidants and ATP-depletion.

### Autophagy-deficient cells are unable to provide protection from oxidant injury and ATP depletion

**Fig 5:**
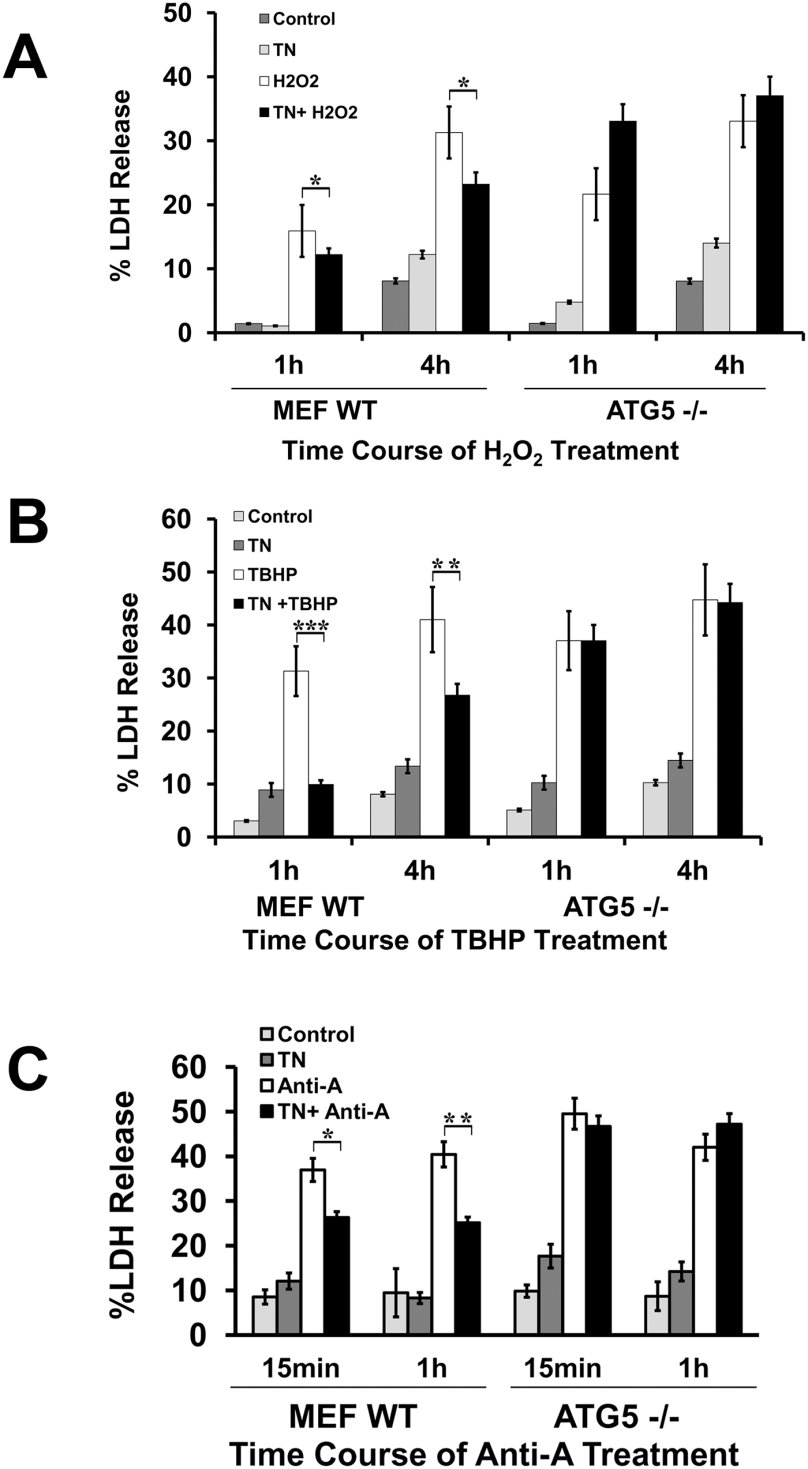
Tunicamycin pretreatment protects Atg5-KO MEFs from oxidant injury and chemical hypoxia compared to wild-type MEFs. **A.** Wild-type and Atg5-KO MEFs were pretreated with 1 μg/ml tunicamycin for 12 h before treatment with 200 μM H_2_O_2_. H_2_O_2_ treatment was given in the time durations indicated. LDH release assay was carried out as an indicator of cytotoxicity. Results are indicated as means of three independent experiments. *P<0.05 compared with H_2_O_2_-treated cells. **B.** Wild-type and Atg5-KO MEFs were pretreated with 1 μg/ml tunicamycin for 12 h before treatment with 50 μM TBHP. TBHP treatment was given in the time durations indicated. LDH release assay was carried out as an indicator of cytotoxicity. Results represent means of three independent experiments. **P<0.01, and ***P <0.001 compared TBHP-treated cells. **C.** Wild-type and Atg5-KO MEFs were pretreated with 1 μg/ml tunicamycin for 12 h before treatment with 10 uM antimycin A. Antimycin A treatment was given in the time durations indicated. LDH release assay was carried out as an indicator of cytotoxicity. Results represent means±SEM of three independent experiments. *P<0.05 and **P<0.01 compared with antimycin A-treated cells.

To further confirm that indeed autophagy is involved in the cytoprotection from oxidants and ATP depletion-induced cell death we used Atg5 (-/-) MEFs. As shown in Fig 5, prior ER stress in Atg5 (-/-) cells was unable to rescue these cells from H_2_O_2_ (Fig 5A), TBHP (Fig 5B), and ATP depletion-mediated cytotoxicity (Fig 5C), suggesting that autophagy induction plays an important role in protection of MEFs against oxidants and ATP depletion.

### ER stress does not impair autophagic flux

**Fig 6:**
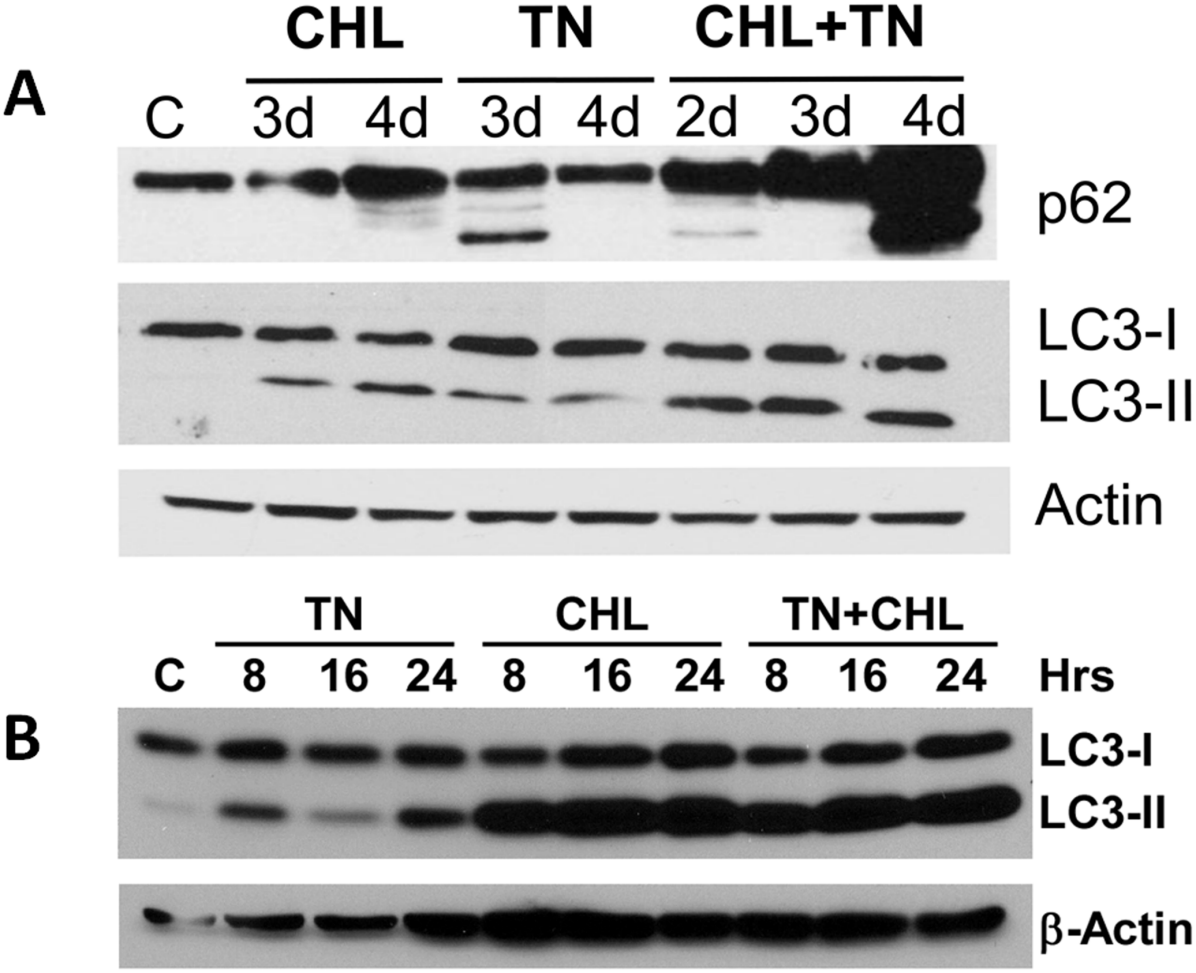
Tunicamycin treatment does not impair autophagic flux in renal cells and kidney. **A.** Kidney tissue obtained from mice treated with 1 mg/kg b.w. tunicamycin alone, 50 mg/kg (b. w.)/day chloroquine alone or tunicamycin + chloroquine for 2d, 3d, and 4d or vehicle (30% DMSO + 70% saline) for 4d was homogenized with lysis buffer containing protease inhibitors. Tissue lysate (50 μg protein) was subjected to western blot analysis using specific antibodies to p62, LC3, and β-actin. β-actin was used as a loading control. The results shown are representative of three independent experiments. **B.** LLC-PK1 cells were treated with 1μg/ml tunicamycin (TN), chloroquine (CHL), and tunicamycin + chloroquine for 8 h, 16h, and 24 h. Cell lysates (50 μg protein) were subjected to western blot using specific antibodies to LC3 and β-actin. β-actin was served as a loading control. The results shown are representative of three independent experiments.

Complete autophagic activity with degradation and clearance of autophagic cargo is referred to as autophagic flux. To determine that autophagic flux was not impaired by ER stress in the kidney, mice were administered the lysosomotropic agent chloroquine in the presence and absence of tunicamycin. Chloroquine inhibits lysosomal function (by increasing intralysosomal pH), blocks autophagosomal clearance, and impairs autophagic flux [35]. During impaired autophagic flux, LC3-II and p62 that are present in the inner membrane of the autophagosome are not degraded and are accumulated [35]. As shown in Fig 6A, chloroquine treatment increased tunicamycin-induced LC3-II and p62 levels in the kidney compared to tunicamycin treated mice. Tunicamycin alone did not increase p62 and LC3-II (Fig 6A) levels suggesting that tunicamycin does not impair the flux of the autophagy pathway. Similarly, in LLC-PK1 cells, chloroquine-treatment markedly increased the production of LC3-II compared to tunicamycin alone (Fig 6B). These findings indicated that tunicamycin-induced autophagic activity proceeds to completion and is not impaired.

### ER stress-induced autophagy ameliorate ischemia-reperfusion injury *in vivo*

**Fig 7:**
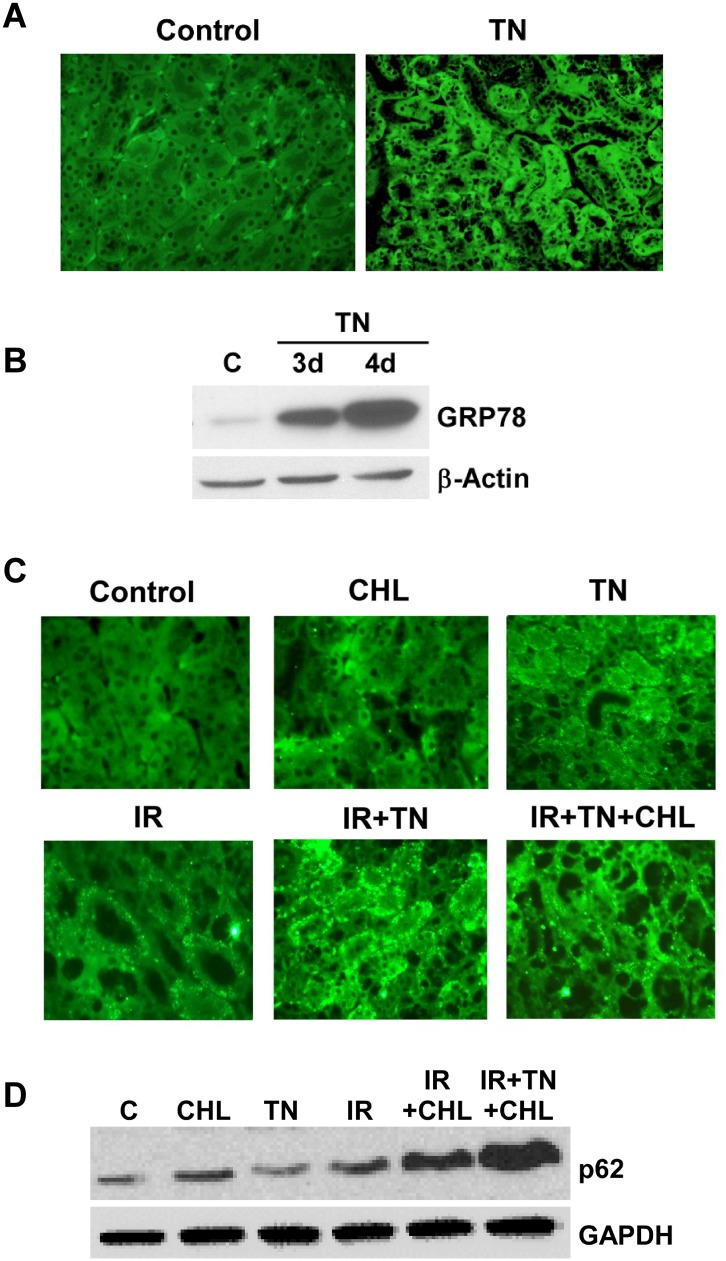
Induction of Grp78 in response to tunicamycin administration and activation of autophagy in the kidney and during renal ischemia-reperfusion injury. **A.** Kidneys from mice treated with tunicamycin (TN) or vehicle (30% DMSO + 70% saline) for 48 hours were processed for immunofluorescence staining as described in Methods. Deparaffinized 5 μm sections were immunostained with a polyclonal rabbit Grp78 antibody followed with an anti-rabbit Alexafluor-488-labeled secondary antibody and images were recorded using an Olympus BX51 fluorescence microscope (40x magnification). The results represent three independent experiments. **B.** Kidney tissue from mice treated with tunicamycin (TN) or vehicle (30% DMSO + 70% saline) for 48 hours was homogenized with lysis buffer containing protease inhibitors. Tissue lysate (50 μg protein) was subjected to western blot analysis using antibodies to Grp78 and β-actin. β-actin was used as a loading control. The results represent three independent experiments. **C.** Mice were treated with tunicamycin or vehicle (30% DMSO +70% saline) for 48 hours and then were subjected to IR. Chloroquine was administered i.p. one hour before IR surgery. Kidneys were processed for immunofluorescent staining as described in Methods. Deparaffinized 5 μm sections were immunostained with polyclonal rabbit anti-LC3 followed with anti-rabbit Alexafluor-488-labeled secondary antibody and pictures recorded on an Olympus BX51 fluorescence microscope at 40x magnification. The results represent three independent experiments. **D.** Mice were treated with tunicamycin or vehicle (30% DMSO +70% saline) for 48 hours and then were subjected to IR. Chloroquine was administered i.p. one hour before IR surgery. Kidney tissue lysates were subjected to western blot analysis using antibody to p62. GAPDH was used as a loading control. The results represent three independent experiments.

**Fig 8:**
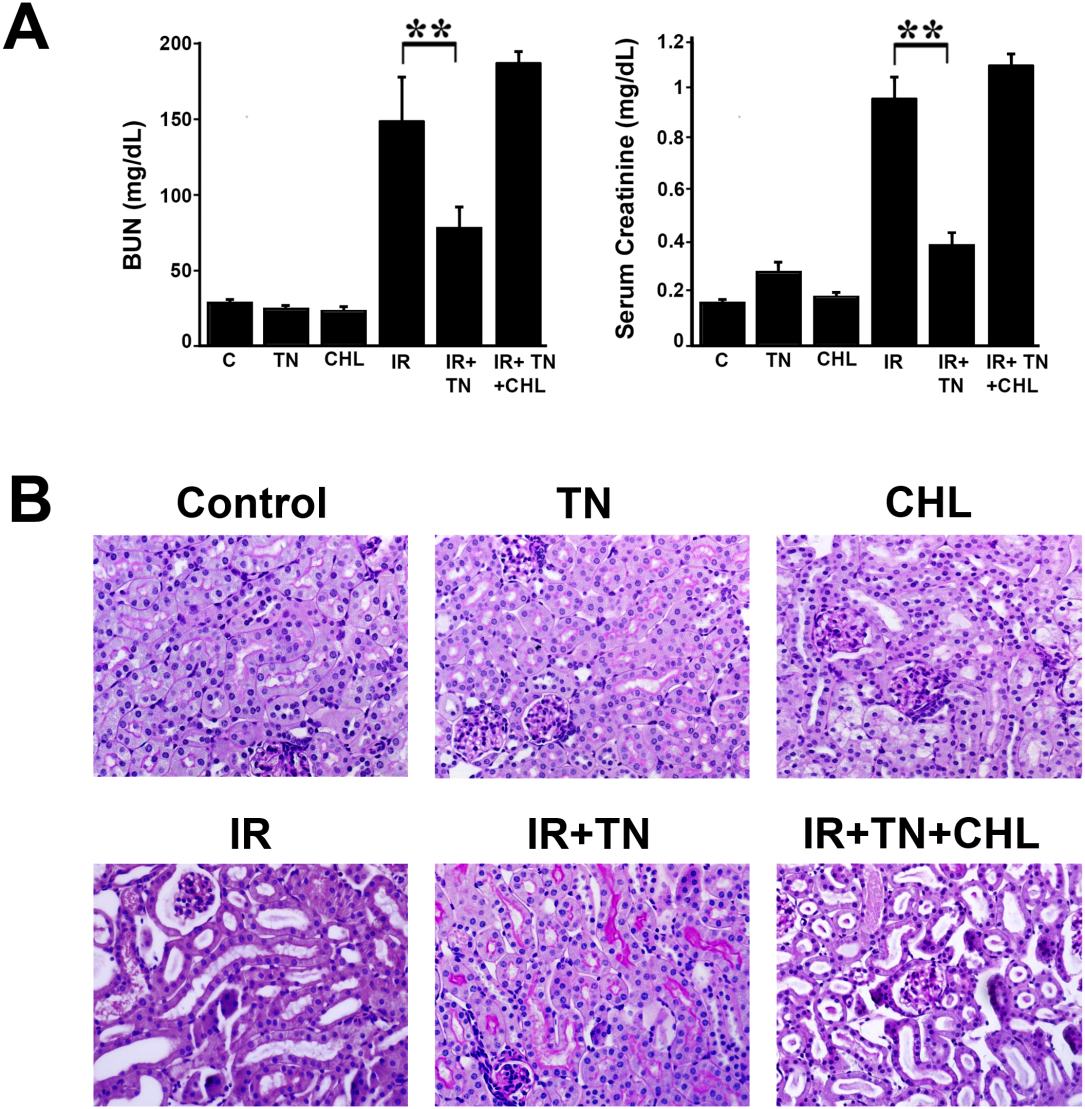
Prior ER stress-induced autophagy ameliorates renal ischemia-reperfusion injury. **A.** Mice were administered 1 mg/kg b.w. tunicamycin (TN) or vehicle (30% DMSO + 70% saline) for 2 d before being subjected to IR injury. For inhibition of autophagy, 50 mg/kg/day chloroquine (CHL) was administered 1 h before ischemia. At the indicated times BUN and serum creatinine were determined. The results are expressed as mean±SEM, n = 5, **P<0.01 for BUN compared with IR alone and **P<0.01for serum creatinine compared with IR alone. **B.** Renal histology in response to vehicle (control), tunicamycin (3d TN), chloroquine (3d CHL), ischemia-reperfusion (IR for 1d), IR+TN, and IR+TN+CHL Kidney sections were stained with PAS staining. The results represent three independent experiments.

Administration of tunicamycin (1.5 mg/kg), that induces significant Grp78 expression, was previously shown to attenuate subsequent renal IR injury [20]. Since a dose of 1 mg/kg tunicamycin also induced Grp78 levels (Fig 7A and 7B) we evaluated whether this dose of tunicamycin also induced autophagy in the kidney tissue. Immunofluorescence staining of kidney sections from tunicamycin-treated mice with an LC3 antibody showed clear punctate staining of LC3 compared to untreated control mice (Fig 7C). The punctate dots were further increased when mice pre-treated with tunicamycin were subjected to IR injury (Fig 7C, IR+TN panel) and this punctate staining appeared to be more or less the same on treatment with chloroquine (Fig 7C, IR+TN+ CHL) due to severe renal injury. Analysis of LC3-II production by immunoblot showed that LC3-II levels in kidney tissue were increased upon administration of tunicamycin suggesting induction of autophagy (Fig 6B). We also examined autophagic flux as measured by p62 levels in the presence and absence of tunicamycin during ischemia-reperfusion injury. As shown in Fig 7D, chloroquine treatment markedly increased both tunicamycin- and IR-induced levels of p62. The level of p62 was slightly increased in IR, however, in the presence of chloroquine the level of p62 increased dramatically. These studies, together with immunostaining with an LC3 antibody, suggest that autophagy is induced during IR injury and that tunicamycin or IR does not appreciably impair autophagic flux. We then examined whether prior activation of ER stress-induced autophagy is able to ameliorate subsequent IR injury. As expected, at 1 d after ischemia, mice showed a marked decline in renal function, as reflected in increased levels of BUN and serum creatinine (Fig 8A). However, prior treatment of mice with tunicamycin displayed a significant decrease in BUN and creatinine levels, indicating ER stress-induced autophagy reduced IR injury. In contrast, inhibition of autophagy by administration of chloroquine worsened renal function (Fig 8A). A previous study has also reported successful inhibition of autophagy in the kidney with chloroquine [36,37,38]. Administration of tunicamycin or chloroquine alone had minimal effect in renal function as evaluated by BUN and creatinine levels. Assessment of histology following ischemia-reperfusion injury revealed tubular damage characterized by acute tubular necrosis, loss of brush-border membranes, tubular dilation, cellular desquamation, and proteinaceous casts of the tubular cells (Fig 8B). Prior treatment of mice with tunicamycin showed significant improvement in renal histology from IR injury (Fig 8B), suggesting marked amelioration of IR injury. Inhibition of autophagy by administration of chloroquine, however, resulted in more severe histological damage (Fig 8B).

## Discussion

In the present study, we have demonstrated that ER stress-induced autophagy plays a key role in providing cytoprotection to renal cells against oxidant- and ATP depletion-induced cell death in cultured cells *in vitro* and renal IR injury *in vivo*. *In vitro* models used in this study are based on the fact that ATP depletion and production of reactive oxygen species are associated with ischemia-reperfusion-induced AKI [39,40,41]. Increased levels of ROS-dependent oxidants are produced and accumulate during the reperfusion phase of IR injury [39,42]. We have demonstrated that both in kidney and cultures RTECs, mild doses of ER stress inducer tunicamycin (1 μg/ml) induced UPR that culminated in the upregulation of the ER chaperone Grp78. Previous studies in mammalian cells have shown that ER stress-induced UPR induction favored survival and facilitated adaptation [43]. Tunicamycin-induced ER stress increased Grp78 levels and attenuated renal dysfunction in renal ischemia-reperfusion injury [20] and provided protection to renal tubular cells against oxidative stress *in vitro* [22,23]. In these studies, however, it was not investigated whether autophagy activation in response to ER stress is involved in the cytoprotection. We observed that induction of ER stress by tunicamycin rapidly activated autophagy in RTECs both in the cell cultures and kidney and inhibition of ER stress-induced autophagy resulted in enhanced cell death and worsened renal injury. Our studies showed that ER stress-mediated activation of autophagy is crucial in affording protection in oxidant and renal ischemia-reperfusion-induced AKI.

Recent studies have provided evidence that ER stress is capable of inducing autophagy in mammalian cells [13,14,15,16]. In addition to its predominant survival role, autophagy also contributes to cell death in some cells depending on the stimulus [5,44]. The cytoprotective role of ER stress-induced autophagy has been reported in capsaicin-mediated ER stress in MCF–7 and MDA-MB–237 cells [45], ER stress in neuroblastoma cells [46], cyclosporine-mediated ER stress in renal tubular cells [16]. ER stress is also known to render cells resistant to apoptosis induced by topoisomerase II inhibitors [47,48]. Autophagic cell death was reported by prolonged ER stress induced by cigarette smoke extract in human umbilical vein endothelial cells [49]. ER stress-induced autophagic cell death was facilitated by upregulation of DAP kinase [50]. In another study, the outcome of ER stress-induced autophagy on cell survival or cell death was shown to be contingent on the type of cells. ER stress-induced autophagy played a pro-survival role in HCT116 colon cancer cells and DU145 prostate cancer cells but a pro-death role in a normal human colon cell line and in non-transformed murine embryonic fibroblasts [44]. Both cannabinoids [51] and glucosamine [52] have been reported to induce ER stress resulting in activation of autophagy-mediated cell death in human glioma cells. ER stress inducers provided protection against 6-hydroxydopamine-induced cytotoxicity in SH-SY5Y cells [53]. Our studies showed that ER stress-induced autophagy protects RTECs from oxidant- and ATP depletion-induced cell death. The pro-survival role of autophagy can be attributed to the ability of autophagy to effectively scavenge damaged and misfolded proteins in the ER [43] to maintain ER function.

Our studies demonstrated that inhibition of ER stress-induced autophagy by 3-MA, wortmannin, chloroquine, or by silencing Atg5 resulted in enhanced activation of caspase–3/7 and apoptosis. On the other hand, inhibition of ER stress-induced autophagy by 3-MA or wortmannin did not result in necrotic cell death. Many previous studies have shown inhibition of ER stress-induced autophagy augmented apoptotic cell death. In W138 lung epithelial fibroblasts cells, inhibition of ER stress-mediated autophagy induced by capsaicin promoted enhanced apoptosis [54]. Inhibition ER stress-induced autophagy in cancer cells [55,56,57,58] and in T Lymphocytes [59] also enhanced caspase activation and apoptosis. However, it is not completely known how inhibition of ER stress-induced autophagy results in apoptotic cell death. Some studies have reported that under irreversible or prolonged ER stress BH3-only proteins of the Bcl–2 family are upregulated at the level of transcription that trigger cytochrome c release and subsequently caspase activation [60]. In tumor cells, inhibition of autophagy has been shown to result in DNA damage under metabolic stress [61] and DNA-damage is well known to activate apoptotic cell death.

Mammalian cells activate the process of autophagy to eliminate damaged proteins and organelles by lysosomal degradation and recycle the resulting amino acids, free sugars, and fatty acids produced for the bioenergetic needs of the cell and for new protein and organelle synthesis. Therefore, if the process of autophagy undergoes completion (normal autophagic flux) it will provide a protective role. Although exact mechanistic role of ER stress-induced autophagy in protection from oxidants, ATP depletion, and IR injury is not known it is likely that autophagy activation provides necessary bioenergetic needs of the cell and eliminate damaged organelles and protein aggregates formed during the injury. Thus the normal flux of the autophagic activity is important in suppressing the cell death pathway. Under certain situations including impairment in the completion of the autophagy process and overdigestion of cytoplasmic contents due to excessive autophagy may lead to autophagic cell death [62]. Our studies demonstrated that tunicamycin-induced autophagy in renal cells undergoes normal autophagic flux. Autophagic flux is an indicator of autophagic activity that involves the dynamic process of autophagosome synthesis, delivery of the autophagic substrates to the lysosome, and degradation of the sequestered substrates by the lysosomal hydrolases. Impaired autophagic flux can result from defective lysosomal function that may retard autophagosome clearance [35]. LC3-II and p62 are present in the inner membrane of the autolysosome and are degraded by the lysosomal hydrolases during the completion of the autophagic flux [63,64]. Thus, impairment of lysosomal function will result in an accumulation of p62 and LC3-II. Our studies demonstrated that treatment of RTECs with the lysosomotropic agents chloroquine or bafilomycin A (that suppress lysosomal function) resulted in further increases of tunicamycin-induced LC3-II and p62 levels suggesting that autophagic flux was not impaired on induction of ER stress.

In response to pathophysiological conditions, ER stress may result from several causes including oxidative stress, energy depletion, or growth factor deprivation resulting in the accumulation of misfolded, damaged, and aggregated proteins. Upon ER stress, cells respond by activating signal transduction pathways such as UPR and autophagy to ensure ER quality control and cellular homeostasis. UPR and associated ER chaperone Grp78 have been shown to predominantly contribute to the induction of autophagy [65,66]. The PERK arm of the UPR pathway has been shown to upregulate Atg12 and convert LC3-I to LC3-II and the IRE1 arm to release beclin1 from the Bcl2 complex to promote autophagy induction. In future studies it will be of interest to study the relationship of autophagy with ATF4 and CHOP in hypoxia/ischemia and oxidant injury. However, at present it is not known which pathway of UPR is involved in the induction of autophagy during IR injury. Thus, mediators of UPR including Grp78 that induce autophagy and pharmacological stimulators of autophagy can be most appropriate for therapeutic interventions in diseases like AKI.

In summary, we have demonstrated that ER stress induced by tunicamycin activated autophagy both in cultured RTECs and in the kidney in a time-dependent manner. Prior ER stress-induced autophagy mediated an adaptive response that afforded protection from oxidative- and ATP-depletion-induced cytotoxicity *in vitro* and ameliorated ischemia-reperfusion injury *in vivo*. Inhibition of autophagy by using pharmacological inhibitors and genetic approaches markedly enhanced oxidative- and ATP-depletion-induced cell death and renal ischemia-reperfusion injury. Our studies demonstrate that ER stress-induced autophagy is a major player in the defense against oxidative- and ATP-depletion-induced cell death *in vitro* and renal ischemia-reperfusion *in vivo*.

## Supporting Information

S1 Fig Flow cytometry of inhibition of ER stress-induced autophagy by pharmacological inhibitors triggered caspase–3/7 activation and apoptotic cell death in LLC-PK1 cells. LLC-PK1 cells were treated with the ER stress inducer tunicamycin (1 μg/ml) for 12 h before treatment with 10 mM 3-MA (**A**) or 50 μg/ml chloroquine (**B**) for varying lengths of time as indicated. The percentage of apoptotic cells under these treatments were determined by FACS analysis using the propidium iodide staining method. Representative flow cytometry data for each treatment and time point are shown. Quantification of 3 independent experiments is depicted in Fig 2E and 2F.
(TIF)
